# Inaugural Dissertation on Circumvolutions of the Umbilical Cord Not Unfrequently Endangering the Life of the Child

**Published:** 1845-04

**Authors:** 


### Art. XVI.

*De circumvolutionibus Funiculi umbilicalis fœtus vitæ haud raro infestis. Dissertatio inauguralis quam consensu et auctoritate gratiosi medicorum ordinis in Academia Heidelbergensi, proposuit* G. A. Mayer, Med.-Chirurg. et artis obst. Doctor.—*Heidelbergiæ*, 1842.

*Inaugural Dissertation on Circumvolutions of the Umbilical Cord not unfrequently endangering the Life of the Child.* By Dr. G. A. Mayer, &c.—*Heidelberg*, 1842. 8vo, pp. 36.

On a former occasion, we have alluded to the care with which many of the inaugural dissertations of the continental universities are written, particularly those from Copenhagen, which for many years have deservedly held so high a rank for the value and originality of their contents; we then took the opportunity of pointing out the great advantages resulting from their being prepared under the immediate auspices of the Professor to whose department the subject of the thesis more immediately belongs. Having received several which have been recently published at the University of Heidelberg, we propose to give a short analysis of them, not merely on account of the information they contain, but if possible to induce the professors and teachers at our own universities and medical schools to follow the example of their continental brethren in this respect.

The thesis which forms the heading of this notice is upon a subject which affords but little scope for novelty, but the author has nevertheless contrived to make it interesting by the quantity of the materials which he has collected about it; it may also be stated that he has had access to the ample records of the Heidelberg Lying-in Hospital, which form statistics of no ordinary extent or value.

In order to estimate the proportion of cases in which circumvolution of the cord occurs, Dr. Mayer has given a tabular arrangement of the labours at the above-mentioned institution, extending over a period of fourteen years. During this time 3587 children were born, and of them 685 had the cord more or less entangled or circumvoluted, giving a proportion of 1 in 5·236. Of these 685 cases, it was round the neck in 594, viz. 482 with a single coil, 100 with the cord twice, 10 with it three times, and 2

with it four times round the neck. In 140 cases it was coiled round other parts of the body, viz. round the feet in 56, round the trunk in 31, the arms in 29, and the shoulders in 25 cases.

It has been a prevailing opinion that entanglement of the cord depended a good deal on its being of unusual length, but the results of Dr. Mayer's observations upon a considerable number of cases do not altogether confirm it. In 656 cases the cord was found once only twelve, and in another case only thirteen inches long; the rest were as follows:

| Cases. | Inches. | Cases. | Inches. | Cases. | Inches. |
|---|---|---|---|---|---|
| 6 | 14 | 73 | 24 | 3 | 34 |
| 18 | 15 | 29 | 25 | 2 | 35 |
| 12 | 16 | 17 | 26 | 7 | 36 |
| 16 | 17 | 60 | 27 | 2 | 37 |
| 70 | 18 | 21 | 28 | 1 | 38 |
| 35 | 19 | 8 | 29 | 1 | 39 |
| 48 | 20 | 25 | 30 | 1 | 41 |
| 78 | 21 | 6 | 31 | 1 | 43 |
| 48 | 22 | 9 | 32 | | |
| 47 | 23 | 15 | 33 | | |

Nevertheless the length of the cord must necessarily be a good deal in proportion to the number of circumvolutions. Thus in 100 cases where it was twice round the child's neck, its length varied from 21 to 39 inches, although in 9 instances it averaged but from 18 to 20 inches, and once only 15. In those cases where it was three times round the neck, it varied from 26 to 36 inches, except in two where it was only 22 inches long. Dr. Mayer has observed that the cases where the cord was of the greatest length were those in which several of the child's limbs were included in the coil.

The symptoms, before or during the early part of labour, of the cord being twisted round some part of the child are extremely doubtful and obscure. The author remarks that a peculiar souffle, synchronous with the fetal heart, and capable of being detected by the stethoscope, may be considered as a proof that the funis is coiled round a portion of the child's body; but he is wrong in giving Professor Naegele, jun., the credit of having first pointed out this fact, as it was not only noticed, but very fully investigated by Dr. Evory Kennedy of Dublin, in his 'Observations on Obstetric Auscultation,' (1833,) under the name of the "funic souffle."

Among the effects upon the child resulting from circumvolution of the cord, a most interesting case by Dr. Jameson is quoted from the American Medical Recorder, (April 1823,) where the abdomen of the fetus had been tightly girded by the cord, and where the upper part of the body had become extraordinarily developed while the lower half was much emaciated; the child was born dead, probably in consequence. The true nature of spontaneous amputation of the limbs before birth, thanks to Dr. Montgomery, is now well understood, and we quote the case as an addition to the many upon this interesting subject which are collecting by some of our obstetric brethren.

Much discrepancy of opinion has existed respecting the injury to the life of the child from circumvolution of the cord. The majority consider that it may be and occasionally is the cause of the child's death.

"In 15 cases of circumvolution Mauriceau observed that, in 3 the child was born dead; La Motte observed 2 out of 6; Smellie 2 out of 5. We feel convinced that the cause of the child's death in these cases must have been attributable to other circumstances. Rœderer says that it is much less frequently a source of danger to the child than at first sight would appear; thus out of 17 cases he observed only 2, where the child's death appeared to be the result of the circumvolution. Haase did not meet with a single instance of a still-born child out of 60 cases of circumvolution of the cord, although in 9 it was drawn so tightly as to require to be cut through." (p. 27.)

In our own practice, with one exception, we cannot call to mind a single instance where the death of the child was clearly attributable to this cause.

From the same ample source of practical information, viz. the Heidelberg Lying-in Hospital, Dr. Mayer has furnished us with some interesting statistics, on the effects of circumvolution of the cord upon the child's life:—

| | | | | Children born dead | |
|---|---|---|---|---|---|
| Date. | Cases of circumvolution of the cord. | Children born alive. | Children born asphyxiated. | from other causes. | from circumvolution of the cord. |
| 1828 | 37 | 34 | 2 | – | 1 |
| 1829 | 39 | 34 | 2 | 2 | 1 |
| 1830 | 45 | 38 | 5 | 2 | – |
| 1831 | 45 | 32 | 5 | 5 | 3 |
| 1832 | 42 | 36 | 5 | 1 | – |
| 1833 | 41 | 31 | 7 | 2 | 1 |
| 1834 | 52 | 38 | 10 | 3 | 1 |
| 1835 | 47 | 38 | 5 | 2 | 2 |
| 1836 | 45 | 41 | 2 | 2 | – |
| 1837 | 51 | 43 | 4 | 1 | 3 |
| 1838 | 37 | 29 | 3 | 4 | – |
| 1839 | 54 | 51 | 1 | – | 2 |
| 1840 | 72 | 55 | 12 | 1 | 4 |
| 1841 | 78 | 64 | 9 | 6 | – |

"The tightness with which the cord is twisted round the neck of the child does not appear on the whole to be so frequent a cause of death as obstruction to the circulation of the cord itself by the pressure of the surrounding parts. Thus when the head presents, the cord may easily be pressed between the neck of the child and the mother's pelvis; in face presentations between the nape of its neck and the occiput, and also when the head is passing under the pubic arch, the cord is in danger of being pressed between the neck and pubic bones. Moreover, where there is a degree of spasmodic contraction of the os uteri when the head has passed through it, or a similar condition of the vagina and os externum, a dangerous amount of pressure may be exerted on the cord when coiled round the child's neck." (p. 29.)

Dr. Mayer gives a case where the child was actually *strangled* by the cord being twisted round its neck, this can only happen where respiration has commenced immediately after the head is born, and where the cord has been drawn tightly by the advance of the child's body; it is a case which can scarcely prove dangerous, if proper assistance be given at the right moment.

